# Martensitic Transformation and Its Microscopic Mechanism of TRIP Duplex Stainless Steel Under Cyclic Loading

**DOI:** 10.3390/ma18102169

**Published:** 2025-05-08

**Authors:** Yixiao Wang, Yi Liu, Hongzhong Wang, Zongyuan Zou, Lei Chen

**Affiliations:** 1 Hubei Modern Manufacturing Quality Engineering Key Laboratory, School of Mechanical Engineering, Hubei University of Technology, Wuhan 430068, China; 2 School of Mechanical Engineering, Wuhan Vocational College of Software and Engineering, Wuhan 430205, China; 3 Key Laboratory of Advanced Forging, Stamping Technology and Science Ministry of Education of China, Yanshan University, Qinhuangdao 066004, China

**Keywords:** TRIP duplex stainless steel, martensitic transformation, cyclic loading, kinetics model

## Abstract

TRIP duplex stainless steels, characterized by high strength and high plasticity, can achieve light-weighting and contribute to reducing fuel consumption and emissions. To further promote the development and application of lightweight metastable duplex stainless steels, the martensitic transformation and the microscopic mechanism of Mn-N alloyed TRIP duplex stainless steel under cyclic loading were investigated. An in situ measurement platform for martensitic transformation under cyclic loading was constructed using an INSTRON 8801 series servo-hydraulic testing machine (Shanghai Instron Test Equipment Trading Co., Ltd., Shanghai, China) and an FMP30 ferrite measuring instrument (Nantong Fischer Testing Instrument Co., Ltd., Nantong, China). The volume fraction of martensitic transformation under symmetrical cyclic loading for different cycles, with strain amplitudes of 0.5%, 0.7%, 0.9%, 1.1%, and 1.3%, was measured. The transformation law of martensite under cyclic loading was analyzed, and a kinetics model for martensitic transformation under cyclic loading was established. Furthermore, the martensitic transformation law, the influence of austenite grain orientation on martensitic transformation, and the microscopic mechanism of martensitic transformation under cyclic loading were analyzed by means of electron back-scattering diffraction (EBSD) and transmission electron microscopy (TEM).

## 1. Introduction

As a lightweight material, metastable austenite duplex stainless steel can be used as an alternative to conventional automotive materials such as cast iron and steel [1]. Cost–benefit analysis and performance evaluation reveal that metastable austenite duplex stainless steel can retain both cost-effectiveness and outstanding performance. In contrast to low-carbon steel, when manufacturing functionally equivalent components, a smaller quantity of metastable austenite duplex stainless steel is needed. This results in an overall reduction in weight, making it a more efficient material choice in terms of both cost and functionality [2]. The weight reduction not only contributes to excellent recyclability and vehicle performance but also improves fuel economy and controls greenhouse gas emissions [3]. At the same time, the additional plasticity and strain-hardening behaviors due to metastable austenite transformation during the plastic deformation can achieve further improvement of the formability of the metastable austenite duplex stainless steel [4]. It especially has great potential application in the field of automobile and rail transit vehicle body production.

Martensitic transformation occurs in metastable austenite duplex stainless steel and brings a transformation-induced plasticity (TRIP) effect. Martensitic transformation can significantly improve the strength and plastic properties of metastable austenite duplex stainless steel. High-strength steel with a yield strength of 1350 MPa has been developed by martensitic transformation [5]. It is the basis of further developing and applying the TRIP duplex stainless steel to clarify the law and mechanism of martensitic transformation. However, the related studies of TRIP duplex stainless steel are mainly limited to that under the uniaxial loading condition [6,7,8]. The martensitic transformation also has an important influence on the cyclic mechanical properties of the transformation steel under cyclic loading [9,10,11]. Through the cyclic loading experiment of TRIP steel under different strain amplitudes, Chen et al. found that the increase in cyclic hardening index is related to the formation of ϵ martensite strengthening phase in metastable austenite under larger strain amplitudes [12]. However, the research on the martensitic transformation of TRIP duplex stainless steel under cyclic loading is rare.

Given the important impact of martensitic transformation on the properties of TRIP steel, the law and mechanism of martensitic transformation have drawn the great interest of researchers [13,14,15]. At present, quantitative descriptions of martensitic transformation with kinetics models of uniaxial tensile phase transformation have been established, including the Sugimoto model [16], the Olson Cohen (OC) model [17], the Gerberich model [18], the Shin model [19], and so on. The kinetics model of martensitic transformation under cyclic loading has gradually been given more attention. Tomita et al. extended the OC model to the condition of cyclic loading and proposed the TI model to describe cyclic loading conditions [20]; Oshida et al. proposed a kinetics model of martensitic transformation under cyclic loading based on autocatalysis [21]; Smaga proposed Smaga model considering the effect of cumulative plastic strain energy on transformation under the cyclic loading [22]. These studies provide a strong theoretical basis for the construction of the kinetics model of martensitic transformation under the cyclic loading of TRIP duplex stainless steel.

In addition to quantitative descriptions of martensitic transformation, the microscopic mechanism of martensitic transformation is also studied by many scholars. Through an in situ tensile test on TRIP duplex stainless steel supplied by Outokumpu Stainless. Tian found that the stability of austenite plays an important role in martensitic transformation, and the stability of austenite is affected by grain orientation, load distribution, and redistribution [23]; Kang et al. studied the effect of grain size on the TRIP effect of duplex stainless steel, and found that the texture has a stronger influence than grain size on TRIP effect [24]. Zhang et al. investigated the microcrack formation in TRIP-assisted duplex stainless steels under large deformation, and the work revealed that microcracks preferentially nucleate at intersections of phase and grain boundaries, and that martensitic transformation hinders slip transfer across interfaces, intensifying local damage [25]. At the same time, Zhang et al. systematically analyzed the contributions of deformation-induced martensitic transformation (DIMT) to strain uniformity and work hardening behavior in TRIP-assisted duplex stainless steel at various temperatures. They revealed that DIMT promotes strain homogeneity in the austenite—martensite system and causes a postponed, rate-dependent stress increment that enhances the hardening capacity of the material [26]. Santos et al. studied the influence of different thermomechanical treatments in the final microstructure of multiphase-steel (0.2%C–1.%Mn–1.5%Si) through morphological characterization of the transformation products. The results show a typical microstructure of multiphase steel (dual-phase) with the increase in the martensite volumetric fraction and hardness values when the steel was submitted to intercritic annealing without reheating after the hot-rolling [27]. Farias et al. tested two AISI304 austenite stainless steels with different initial states at room temperature under low cycle fatigue with different strain amplitudes. The results show that the cyclic-induced volume fraction of martensitic transformation largely depends on the initial state of the material, and the stacking faults and micro-twins in the initial state of the material may lead to martensitic nucleation [28]. The nucleation of TRIP duplex stainless steel is an important part of the microscopic mechanism of martensitic transformation. At present, research on the nucleation mechanism of martensite focuses on the conditions of uniaxial tensile. Through uniaxial tensile test and EBSD analysis, Xu et al. studied the nucleation mechanism of martensitic transformation in 304HC stainless steel wire. The nucleation position of martensitic transformation is at the interface between twin grains and martensite. $\alpha^{\prime }$ martensite nucleates at the shear band and accumulates to form martensite laths [29]. Choi et al. show that the growth rate of deformation-induced martensite nucleation at the austenite grain boundary and twin grains is larger than that nucleated in austenite grains [30]. These studies have great significance in revealing the microscopic mechanism of martensitic transformation. However, although significant efforts have been made to investigate martensitic transformation in TRIP steels under monotonic or uniaxial loading conditions, studies focusing on cyclic loading—particularly in Mn-N alloyed duplex stainless steels—remain scarce. In the latest research, Wang et al. investigated a Mn–N alloyed lean duplex stainless steel and revealed that a positive strain rate dependence can be achieved by promoting the γ → ϵ → $\alpha^{\prime }$ transformation pathway under high-speed deformation, which effectively suppresses early martensitic cracking and enhances the TRIP effect [31]. They also researched the strain rate-dependent deformation behavior of Mn–N alloyed lean duplex stainless steel and demonstrated that increased strain rate suppresses the γ → ϵ → $\alpha^{\prime }$ transformation sequence due to adiabatic heating, which raises the stacking fault energy and delays martensite nucleation [32]. Hao et al. systematically studied the low-cycle fatigue (LCF) behavior of Mn–N bearing duplex stainless steel. They established a unified transformation kinetics model based on accumulated plastic strain and identified $\alpha^{\prime }$ martensitic transformation as a key factor in fatigue crack nucleation and propagation, especially near phase interfaces under strain-controlled cyclic loading [33]. Moreover, existing works lack systematic kinetic modeling under cyclic loading and fail to uncover the underlying microscopic mechanisms during the transformation process.

In this work, the martensitic transformation of a new type of TRIP duplex stainless steel with Mn-N instead of Ni under cyclic loading was studied. Mn and N were selected as primary alloying elements to replace Ni, reducing material costs while maintaining austenite stability. Ni is a well-known austenite stabilizer that improves the ductility and toughness of austenitic stainless steels, Mn and N play different roles. Mn is an austenite stabilizer that reduces the stability of austenite compared to Ni, thus promoting martensitic transformation under mechanical deformation. N, on the other hand, is a solid solution strengthening element that not only enhances the strength of austenite but also helps in stabilizing the austenite phase at lower temperatures. Mn enhances strain hardening, and N improves yield strength and pitting resistance, aligning with ASTM A240 specifications [34]. In TRIP steels, Mn and N together enhance the transformation-induced plasticity (TRIP) effect by improving the driving force for martensitic transformation. Specifically, Mn increases the transformation kinetics, while N increases the strength of the retained austenite and contributes to the fine grain structure, facilitating the martensitic transformation under cyclic loading. Therefore, the choice of Mn-N as a substitute for Ni in duplex stainless steels is expected to promote a more pronounced TRIP effect under cyclic loading, leading to improved mechanical properties such as strength and ductility. This design targets automotive components (e.g., chassis, crash-resistant structures) where cyclic loading resistance and weight reduction are prioritized. By virtue of the in situ measurement platform for martensitic transformation, symmetrical cyclic loading tests at various strain amplitudes were carried out, and the volume fraction of martensitic transformation under different cycles was measured. The work further introduces a kinetics model that integrates both the TI and Smaga formulations to describe transformation dynamics under cyclic conditions, achieving strong agreement with experimental data. The microstructure of TRIP duplex stainless steel under cyclic loading was characterized by TEM and scanning electron microscopy combined with EBSD, and the microscopic mechanism of martensitic transformation under cyclic loading for TRIP duplex stainless steel was studied.

## 2. Material and Methods

The experimental steel used in this paper is Mn-N alloyed economical duplex stainless steel. First, set each component and smelt to obtain the experimental steel. Then, a spectrometer is used to measure and obtain the chemical composition list of the experimental steel for verification. The main chemical constituents of the experimental steel are shown in Table 1.

**Figure 1:**
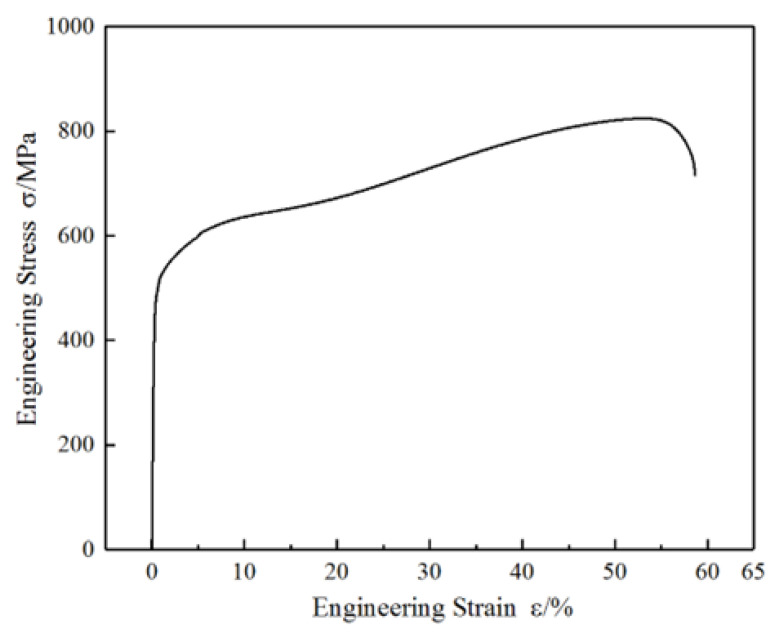
Engineering stress–strain curve under uniaxial tensile.

The relationship between engineering stress and strain of the experimental steel was measured, as shown in Figure 1. The yield strength of the experimental steel is 481.8 MPa, the tensile strength is 824.6 MPa, the fracture elongation is 58.7%, the elastic modulus is 226.1 GPa, there is no yield platform, and the yield strain is about 0.5%. In comparison with conventional duplex stainless steels, the experimental Mn–N alloyed duplex stainless steel demonstrates significantly enhanced mechanical properties, particularly in terms of elastic modulus and elongation. This improvement can be primarily attributed to the transformation-induced plasticity (TRIP) effect derived from the presence of metastable austenite in the alloy. Specifically, by replacing costly Ni with Mn and N, the stability of the austenite phase is intentionally reduced, rendering it metastable under mechanical loading. During cyclic deformation, this metastable austenite undergoes stress- or strain-induced martensitic transformation, which activates the TRIP effect. The progressive transformation enhances strain hardening and delays necking, thereby improving both ductility and the work hardening rate. Furthermore, nitrogen contributes to solid solution strengthening and promotes grain refinement, while manganese facilitates the formation of a finer austenitic structure and increases the driving force for martensitic transformation. Collectively, these factors synergistically result in the observed increase in elastic modulus and elongation compared to conventional duplex stainless steels. With the increase in deformation, the engineering stress–strain curve shows an obvious secondary increase. The work hardening rate curve first decreases, then increases to the peak and then decreases again, showing the “three-stage” hardening characteristics. It can be inferred from this that the metastable austenite phase in the test steel underwent deformation-induced martensitic transformation during the deformation process, generating the TRIP effect.

**Figure 2:**
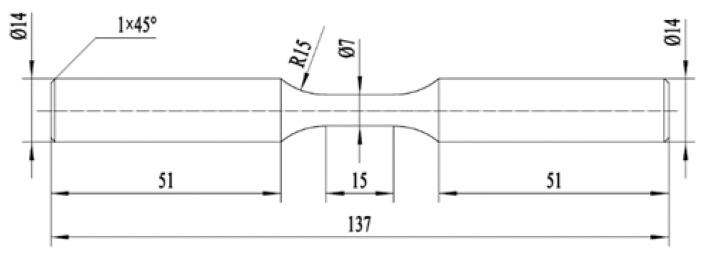
The specimen size of the cyclic loading test.

The experimental steel specimen with a circular section used for the cyclic loading test is shown in Figure 2 concerning ASTM E606/E606M-2012 [35].

**Figure 3:**
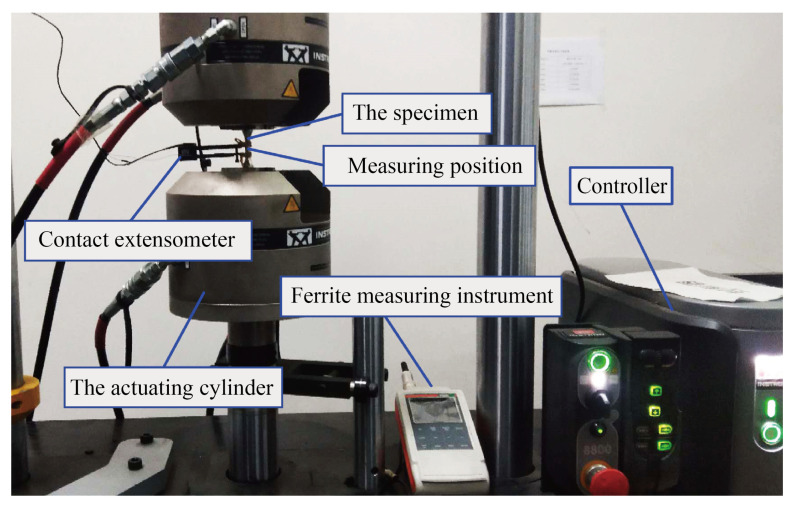
In situ analysis platform for martensitic transformation.

**Figure 4:**
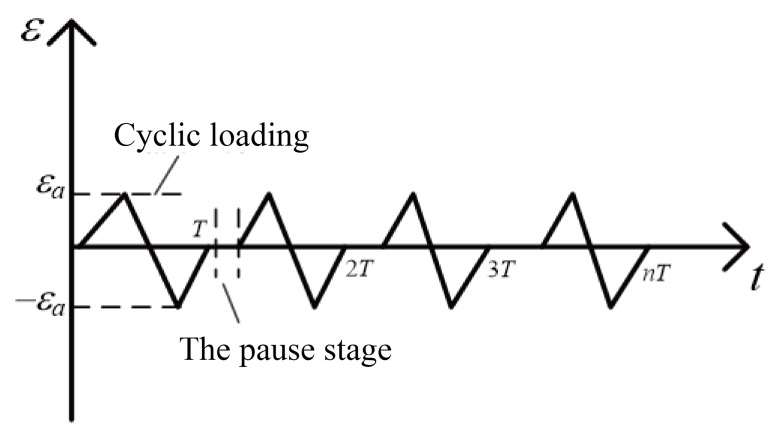
Waveform of cyclic loading.

The Fischer FMP30 ferrite measuring instrument (Nantong Fischer Testing Instrument Co., Ltd., Nantong, China) is used to accurately measure the transformation of $\alpha^{\prime }$ martensite by measuring the volume fraction of the ferromagnet, that is the body-centered cubic (BCC) structure. In this study, the Fischer FMP30 ferrite measuring instrument and Instron 8801 series servo-hydraulic testing machine (Shanghai Instron Test Equipment Trading Co., Ltd., Shanghai, China) are combined to build an in situ measurement platform for measuring the transformation of $\alpha^{\prime }$ martensite under cyclic loading. By reasonably compiling the cyclic loading control program of the servo-hydraulic testing machine, the ferrite measuring instrument can be used to measure the volume fraction of martensite many times in given cycles. The in situ measurement platform for martensitic transformation is shown in Figure 3. The loading control path of the servo-hydraulic testing machine is shown in Figure 4. The loading control path is divided into two periods, namely the cyclic loading stage and the pause stage (the stage for measuring the volume fraction of strain-induced martensite).

**Figure 5:**
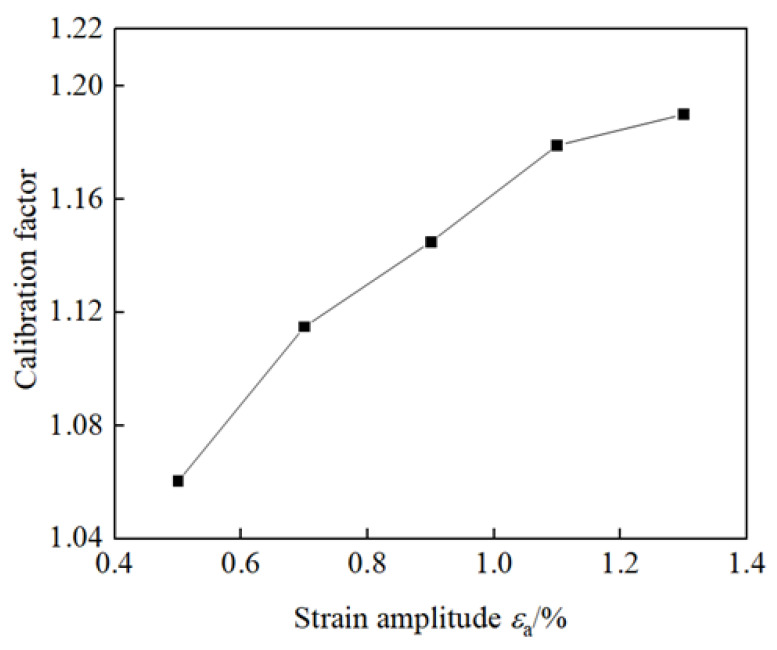
Calibration factors for each strain amplitude.

It is known that the measurement accuracy of the ferrite measuring instrument is affected by the geometric size of the specimen and mechanical load. In this paper, the Fisher FMP30 ferrite measuring instrument is used based on the principle of magnetic induction to accurately measure the ferromagnetic content in the sample by measuring the induced voltage. Ferrite in TRIP duplex stainless steel is ferromagnetic, and the $\alpha^{\prime }$ martensite produced by phase transformation also has ferromagnetism. Therefore, by measuring the change in ferromagnetic content with a ferrite measuring instrument, the transformation amount of the phase $\alpha^{\prime }$ martensite can be obtained [36]. Talonen J et al. [37] studied the measurement methods of $\alpha^{\prime }$ martensite content, including magnetic induction method, X-ray diffraction method and optical metallographic method, etc., and found that there was a linear relationship between the $\alpha^{\prime }$ martensite content measured by the magnetic induction method and the true content. The accurate transformation amount of $\alpha^{\prime }$ martensite can be obtained by calibrating the measured values of the Fisher FMP30 ferrite measuring instrument. In this study, based on the structural form and size of the sample, this procedure was repeated for each strain amplitude using specimens of identical geometry to eliminate dimensional effects. The difference between pre- and post-holding values allowed for correction of magnetic measurement distortions induced by residual stresses. And in accordance with the calibration factors given in the “Fisher FMP30 Ferrite Measuring Instrument User Manual”, calibration was performed multiple times to ensure repeatability and consistency, the calibration factors of the martensite transformation amount in this experiment were determined, the value of 1.16 is taken as the calibration factor in this test. In addition, different calibration factors of mechanical load are needed for different strain amplitudes. In this study, the calibration factors of various strain amplitudes were obtained by the measured values of specimens with different strain amplitudes before and after holding (not loaded), as shown in Figure 5. In this study, the volume fraction of martensite was measured under symmetrical strain cyclic loading with strain amplitudes of 0.5%, 0.7%, 0.9%, 1.1%, and 1.3%. The selected strain amplitudes ranging from 0.5% to 1.3% were chosen to cover the typical range of cyclic deformation observed in TRIP steels under practical loading conditions. Strain amplitudes below 0.5% are generally insufficient to induce significant martensitic transformation, while amplitudes above 1.3% may lead to excessive plastic deformation or failure in the material. This range allows for the investigation of the gradual evolution of martensitic transformation with increasing strain amplitude, providing a comprehensive understanding of the material’s mechanical response and phase transformation behavior under cyclic loading. The measured ferrite content, which reflects the volume fraction of strain-induced $\alpha^{\prime }$ martensite, is influenced by both the geometry of the specimen and the mechanical loading state. As the strain amplitude increases, the applied stress and strain energy density during cyclic loading also rise, leading to enhanced magnetic permeability changes in the transformed regions. This necessitates the use of specific calibration factors for different strain amplitudes to correct for magnetic field distortion caused by mechanical stress.

## 3. Results and Discussion

### 3.1. In Situ Measurement of Martensitic Transformation Under Cyclic Loading

**Figure 6:**
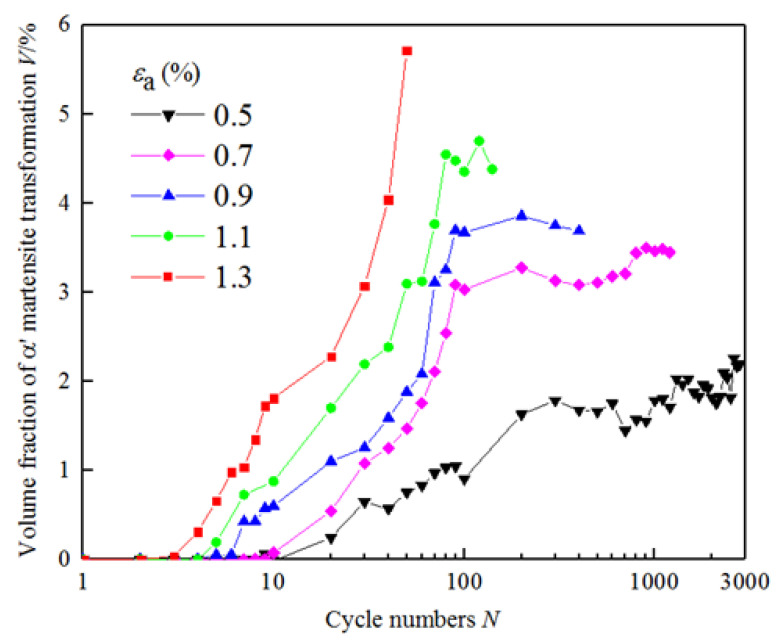
The volume fraction of $\alpha^{\prime }$ martensitic transformation with cycles under different strain amplitudes.

Figure 6 shows the curve of $\alpha^{\prime }$ martensitic transformation of the experimental steel with cycles, which is under different strain amplitudes. The results show that the martensitic transformation under cyclic loading of the experimental steel has the following characteristics: under cyclic loading, martensitic transformation of metastable austenite occurs even at a low strain amplitude of 0.5%. The metastable austenite does not completely transform into martensite under the cyclic loading, and most metastable austenite is retained. The martensitic transformation increases with the increase in strain amplitude at the same cycle. The larger the strain amplitude is, the earlier the martensitic transformation occurs. In the end, the transformation tends to saturation with cycles, and the martensitic transformation mainly occurs in the initial 100 cycles.

### 3.2. A Kinetics Model of Martensitic Transformation Under Cyclic Loading

By combining the TI model [20] with the Smaga model [22], a kinetics model describing the transformation behavior of retained austenite under cyclic loading was developed, as shown in Equation (1). According to the volume fraction of martensite measured in the experimental steel and based on the kinetics model of martensitic transformation under the cyclic loading with TI model and Smaga model, the kinetics model of martensitic transformation under cyclic loading of the experiment steel is established:

(1) $$f_{M}=-k\varepsilon_{a}{\left[1-exp\left(-\Phi S_{p}\right)\right]}^{m}$$

where *k*, Φ, and *m* are material parameters, $\epsilon_{a}$ is the strain amplitude, *S_p_* is the true cumulative plastic strain, and $f_{M}$ is the volume fraction of martensite.

(2) $$S_{p}=4N\varepsilon_{a}^{p}$$

where $\epsilon_{a}^{p}$ is the true plastic strain amplitude at half of the fatigue life (0.5 $N_{f}$) and *N* is the cycle number (C).

(3) $$\varepsilon_{a}^{p}=\varepsilon_{a}-\frac{\sigma_{a}}{E}$$

where $\sigma_{a}$ is the stress amplitude (MPa) at half the fatigue life (0.5 $N_{f}$), and E is the elastic modulus (MPa).

By introducing Equations (2) and (3) into Equation (1), it can be concluded that

(4) $$f_{M}=-k\varepsilon_{a}{\left\{1-exp\left[-4\Phi N\left(\varepsilon_{a}-\frac{\sigma_{a}}{E}\right)\right]\right\}}^{m}$$

According to Equation (4), the kinetics model parameters are determined by fitting the measured data of $\alpha^{\prime }$ martensitic transformation of the experimental steel under the strain amplitude of 1.1%, as shown in Table 2.

The values of material constants in Table 2 are brought into Equation (4), and the kinetics model of martensitic transformation under the cyclic loading is obtained as follows:

(5) $$f_{M}=4\varepsilon_{a}{\left[1-exp\left(-2.008N\varepsilon_{a}^{p}\right)\right]}^{1.2}$$

**Figure 7:**
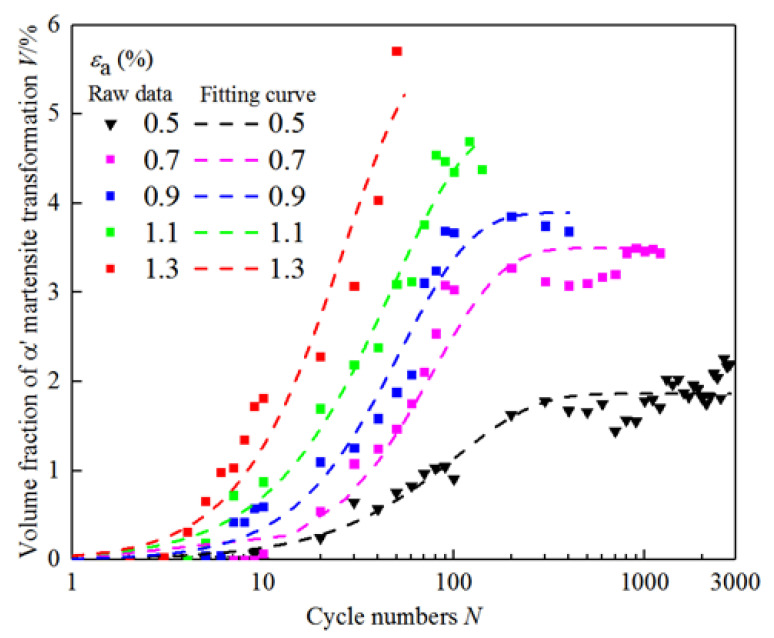
Raw data and fitting curve of $\alpha^{\prime }$ martensitic transformation.

The curves of the above kinetics model under different strain amplitudes are shown in Figure 7. Compared with previous studies on transformation kinetics in TRIP steels under cyclic loading, the proposed model achieves comparable or superior prediction accuracy. For example, Smaga et al. [22] reported transformation prediction errors within 10% for AISI 304 under low-cycle fatigue, while Tomita et al. [20] applied the TI model to carbon steel with a lower strain range. Our model, combining TI and Smaga formulations, achieves over 90% correlation with measured values across a broader strain amplitude range (0.5% to 1.3%), demonstrating better generalizability and reliability for Mn–N alloyed duplex stainless steels. This confirms the applicability of the proposed model to multi-phase TRIP systems beyond single-phase austenitic steels.

**Figure 8:**
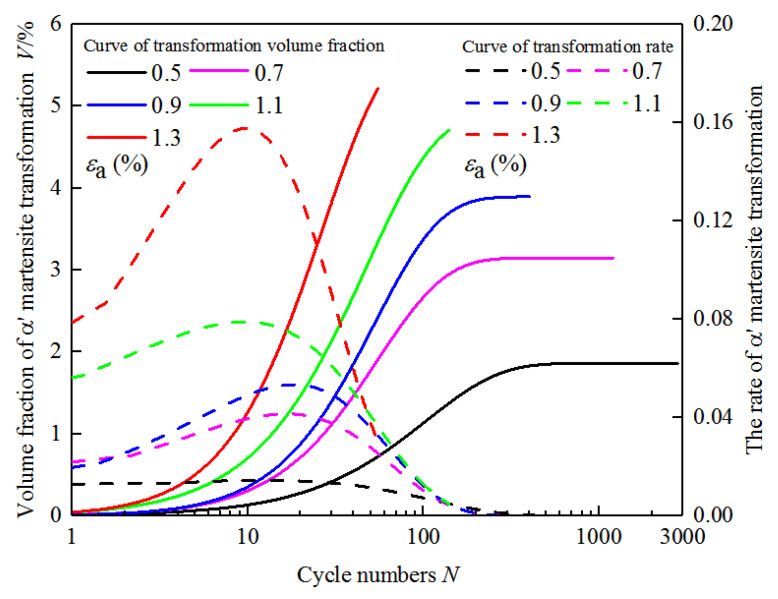
The volume fraction and rate curves of $\alpha^{\prime }$ martensitic transformation.

By deriving the kinetics curve of martensitic transformation under the cyclic loading, the curve of martensitic transformation rate with cycles is obtained, as shown in Figure 8. It can be seen from the figure that the martensitic transformation rate of the experimental steel is not constant under the cyclic loading. The transformation rate gradually increases at the initial stage of cyclic deformation. And after reaching the peak, it gradually decreases to zero. Moreover, at the initial stage of cyclic deformation, the larger the strain amplitude is, the larger the martensitic transformation rate is, and the earlier it reaches the peak. This behavior is attributed to the increased mechanical driving force for transformation at higher strain levels, which facilitates the formation and multiplication of transformation-induced defects such as stacking faults, shear bands, and dislocation structures, thereby promoting martensitic nucleation and growth. In contrast, lower strain amplitudes result in slower transformation kinetics and a lower final volume fraction of martensite, as the mechanical energy available is insufficient to activate a large number of nucleation sites. Moreover, the metastable austenite in Mn-N alloyed steels exhibits varying degrees of stability depending on local grain orientation and composition. At lower strain levels, the retained austenite remains largely untransformed due to its relatively higher stability, particularly in grains oriented along <111> or those strengthened by interstitial N atoms. Therefore, the difference in transformation behavior across strain amplitudes is a combined result of deformation-induced defect evolution, austenite phase stability influenced by Mn-N alloying, and the thermomechanical threshold required to initiate the γ→$\alpha^{\prime }$ transformation. It is also worth noting that, although the martensitic transformation rate reaches its peak around the 15th cycle and gradually decreases thereafter, the volume fraction of martensite continues to increase with further cycling (N > 15C). This behavior reflects the classic transformation kinetics where nucleation is dominant in the early stages, followed by a growth-controlled regime. In the initial cycles, rapid strain localization and high dislocation densities lead to abundant nucleation of $\alpha^{\prime }$ martensite, especially at grain boundaries and shear band intersections. As cycling progresses, the easily transformed regions of metastable austenite are progressively consumed, and the remaining austenite becomes more stable either due to orientation or local strengthening effects. Consequently, the number of new nucleation sites diminishes, and the transformation rate decreases. Nevertheless, residual austenite continues to transform gradually under accumulated plastic strain, albeit at a slower rate, leading to a continued increase in the martensite volume fraction.

### 3.3. The Microscopic Mechanism of Martensitic Transformation Under Cyclic Loading

In this study, quasi in situ cyclic test with a strain amplitude of 1.1% was carried out to study the microscopic mechanism of martensitic transformation under cyclic loading. The five cycles selected were 0, 5, 15, 50, and 150 (fatigue), respectively. The specimens for microscopic observation were cut from the center position of the gauge section.

#### 3.3.1. EBSD Analysis

**Figure 9:**
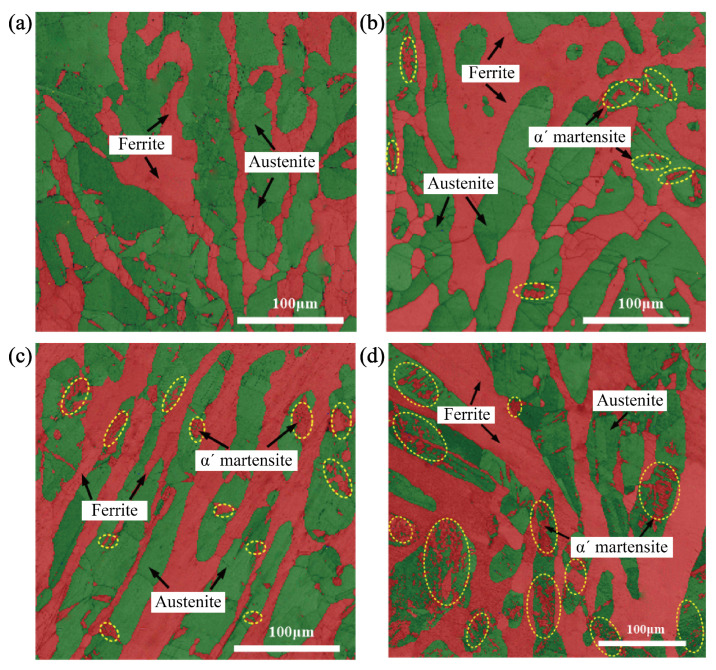
EBSD analysis diagram under different cycles showing. (**a**) Initial stage. (**b**) The 15th cycle. (**c**) The 50th cycle. (**d**) Fatigue state at the 150th cycle. As the cycle period increases continuously, the circled part in the figure keeps changing.

Figure 9 is the EBSD analysis diagram of the experimental steel at the initial state and different cycle numbers. Due to the large scanning step set in the test, it belongs to the micron level, while ϵ martensite belongs to the nanometer level, and ϵ martensite cannot be identified. The observation of ϵ martensite is analyzed by TEM in the following. Figure 9a shows the microstructure at the initial state after solution treatment at 1050 °C, with body-centered cubic (BCC) structure in red and face-centered cubic (FCC) structure in green. It can be seen from the EBSD images that the overall morphology and orientation of the two phases are not obvious, and the austenite with FCC structure is distributed in the ferrite matrix with BCC structure as an island. There are also a few red areas in austenite, which can be divided into two types. The first type is distributed at the intersection of austenite grain boundaries with a strip shape; the second one is distributed in the austenite grain, which is wedge-shaped. These red areas may be $\alpha^{\prime }$ martensite with the BCC structure produced during the solution treatment.

In Figure 9, the ferrite and the $\alpha^{\prime }$ martensite are both BCC and so both are marked in red. The $\alpha^{\prime }$ martensite is produced by austenite, which is in the austenite. It can be seen from the figure that the strain-induced $\alpha^{\prime }$ martensite is in the shape of a sieve, as shown in the yellow dotted line in Figure 9. It can be seen from Figure 9b that only a small portion of austenite grains can produce martensite at the 15th cycle, which indicates that the austenite has poor stability and is easy to transform into martensite, but it has a low volume fraction. According to Figure 8, the volume fraction and the rate of martensitic transformation change with cycles, and the volume fraction of martensitic transformation is less in the range of 0–15 cycles. But the transformation rate increases rapidly, and it reaches the peak at the 15th cycle; $\alpha^{\prime }$ martensite still increases with the increase in cycles (N > 15C). Compared with the EBSD diagram at the 15th cycle, the EBSD diagram at the 50th cycle shows that the amount of martensite increased. The martensite is flaky, and the austenite tends to be sieve-like. This phenomenon is more obvious in the fatigued specimen. According to Figure 8, the rate of martensitic transformation gradually decreases after 15 cycles, but the volume fraction of martensite still increases gradually and reaches the maximum at fatigue. The transformation rate drops to zero at the same time.

Martensitic transformation is affected not only by the stability of austenite but also by the strain of the parent phase. It can be seen from Figure 9 that the transformation of austenite does not occur uniformly under cyclic loading. Martensitic transformation occurs only in a portion of austenite at the initial cyclic stage. With the increase in austenite strain, martensitic transformation occurs in a larger portion of austenite at the later stage of cyclic loading. It can be seen from the figure that, in addition to a small portion of martensite in austenite grains, martensite is also produced at austenite grain boundaries at the initial cyclic stage. This is because there are stress concentrations at grain boundaries, where much more strain is obtained preferentially, thus reaching the starting point of martensitic transformation preferentially, and then martensitic transformation occurs.

**Figure 10:**
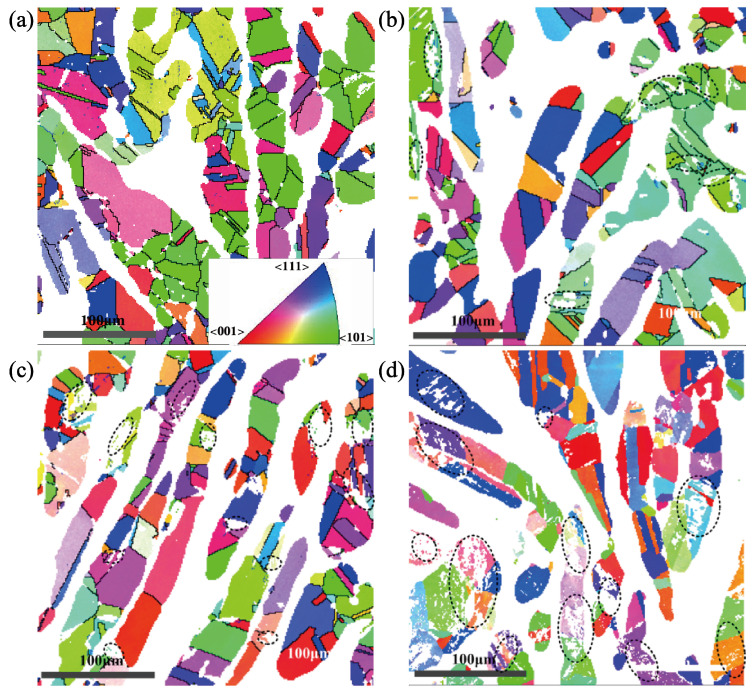
Orientation of austenite grains at different cycles showing. (**a**) Initial stage. (**b**) The 15th cycle. (**c**) The 50th cycle. (**d**) Fatigue state at the 150th cycle.

Previous studies have shown that the transformation of austenite is related to the orientation of austenite grains, which is relative to strain direction under uniaxial tensile. As shown in Figure 10 combined with Figure 9, for the austenite grain orientation in the experimental steel under the cyclic loading with a strain amplitude of 1.1%, the martensitic transformation mainly occurs in the austenite with grain orientation <101> after 15 cycles; The austenite with grain orientations <001> and <101> produce martensitic transformation after 50 cycles, and the austenite with grain orientations <001>, <101>, and <111> both produce martensitic transformation at fatigue. Therefore, with the increase in cumulative plastic strain under the cyclic loading, the austenite grain orientations corresponding to the degree of difficulty for martensitic transformation are <101>, <001>, <111> from easy to difficult.

It is found that the austenite with grain orientation <101>is much more at the original state by analyzing the grain orientation diagram. Thus, more austenite grains are prone to transform into martensite, and the martensitic transformation rate is larger at the initial cyclic stage; the more stable retained austenite with grain orientations <101> and <111> are not easy to transform into martensite. Thus, the martensitic transformation rate begins to decrease, as shown in Figure 10, with a strain amplitude of 1.1%.

#### 3.3.2. TEM Analysis

**Figure 11:**
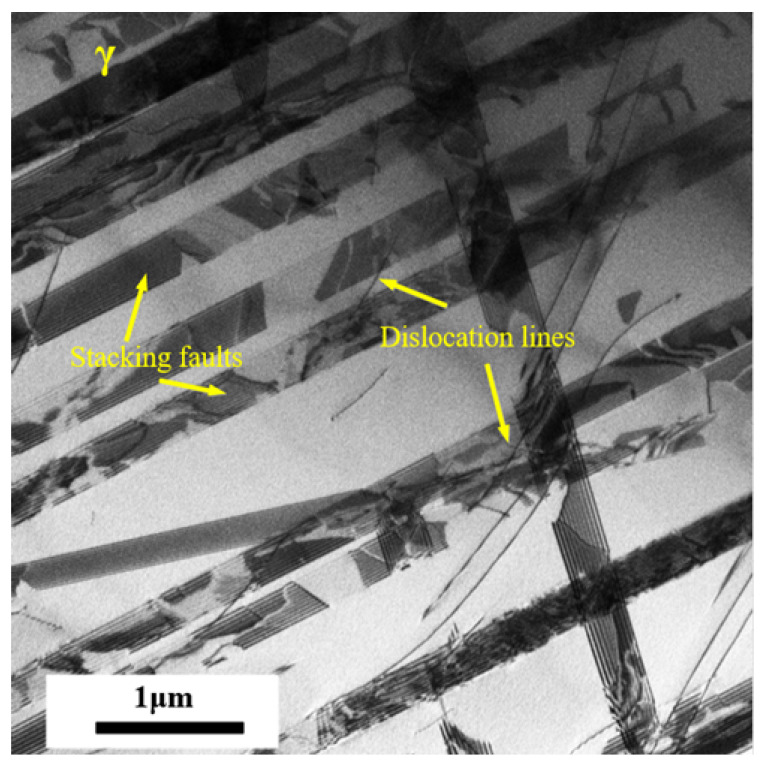
A TEM photo of the original austenite.

To further explore the nucleation of martensitic transformation in experimental steel under cyclic loading, TEM analysis was carried out on the experimental steel with different cycles with a strain amplitude of 1.1%. For the nucleation of martensitic transformation, previous studies have shown that the nucleation position of martensitic transformation may include the intersection of shear bands, the intersection of shear bands and grain boundaries, the intersection of grain boundaries, and the position of the shear band, etc. [38]. Figure 11 shows the microstructure of the austenite in the original state. There are many stacking faults and a small number of dislocation lines. It can be inferred that these stacking faults provide nucleation positions for the subsequent formation of ϵ martensite and $\alpha^{\prime }$ martensite.

**Figure 12:**
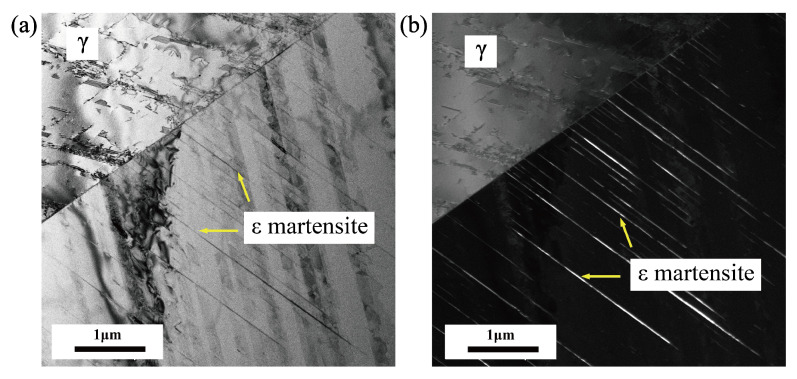
TEM bright and dark field diagrams of austenite at the fifth cycle showing. (**a**) The bright field diagram. (**b**) The dark field diagram.

Figure 12 shows the microstructure of the experimental steel at the fifth cycle. The deformed band structure running through the whole austenite grain is parallel distributed in the austenite, which is white in the dark field diagram, indicating that these structures are different from the structure of the matrix, and they are the newly formed phase. According to the relevant literature, ϵ martensite is a thin strip, so it can be inferred that the fine strip structure is ϵ martensite. Compared with the original state, austenite grains produce phase transformation and ϵ martensite. In addition, compared with the TEM diagram of the original state in Figure 11, the stacking fault density is also significantly reduced. This is because the winding and superposition of the stacking fault is the intermediate step of transformation from austenite to ϵ martensite, and the density of the stacking fault decreases due to the formation of ϵ martensite [39].

**Figure 13:**
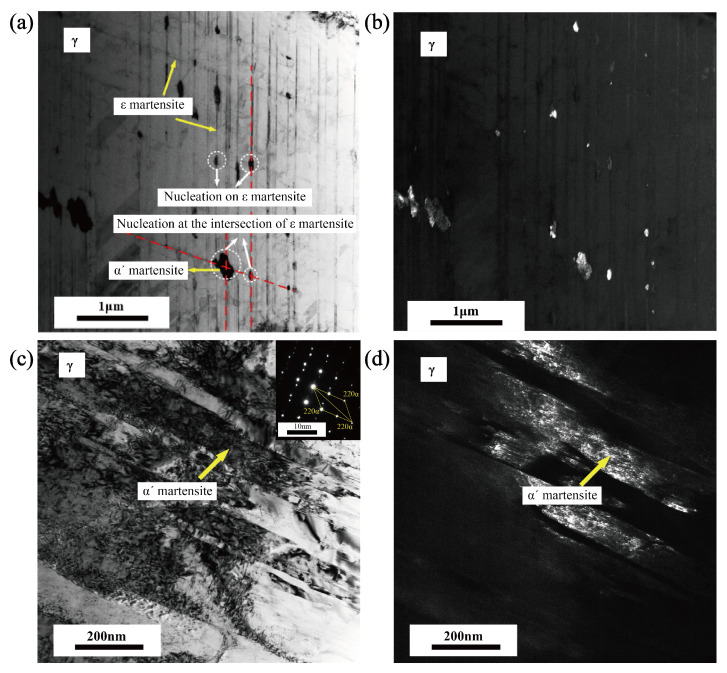
TEM analysis diagrams at fatigue showing. (**a**) TEM bright-field diagram (ϵ → $\alpha^{\prime }$). (**b**) TEM dark-field diagram (ϵ → $\alpha^{\prime }$). (**c**) TEM bright-field diagram (γ → $\alpha^{\prime }$). (**d**) TEM dark-field diagram (γ → $\alpha^{\prime }$).

Figure 13 presents the bright-field and dark-field transmission electron microscopy (TEM) images of the austenite microstructure after cyclic loading, revealing the microscopic features of martensitic transformation. It can be seen from Figure 13a,b that, in addition to the strip structures (ϵ martensite), there are also block structures in austenite. The comparison between the bright and dark field diagrams shows that the strip structures are different from the block structures. Consequently, it can be inferred that the block structures are $\alpha^{\prime }$ martensite. In addition, it can be seen from the figure that a portion of $\alpha^{\prime }$ martensite is produced at the intersection of ϵ martensite, and the other portion produced on ϵ martensite. Figure 13c,d are the bright and dark field diagrams of austenite at another location. The lamellar structure in austenite grain can be identified as $\alpha^{\prime }$ martensite by diffraction pattern, which means the $\alpha^{\prime }$ martensite is the martensitic transformation that is produced in austenite grain. Based on these microstructural observations, three distinct microscopic mechanisms for $\alpha^{\prime }$ martensitic transformation under cyclic loading can be established: (1) A sequential transformation where austenite first transforms into ϵ martensite, and then $\alpha^{\prime }$ martensite nucleates at the intersection of ϵ martensite plates. (2) A similar γ → ϵ → $\alpha^{\prime }$ pathway, with $\alpha^{\prime }$ martensite nucleating on the surface or boundary of ϵ martensite. (3) A direct γ → $\alpha^{\prime }$ transformation that occurs independently within the austenite grain, driven by strain localization and high dislocation density induced by cyclic deformation. These results indicate that cyclic loading promotes multiple martensitic transformation pathways, depending on the local microstructural stress state, slip activity, and ϵ martensite distribution. The co-existence of these mechanisms highlights the complex interplay between metastable austenite stability, deformation-induced defects, and interphase interactions during fatigue loading.

The observed nucleation mechanisms of $\alpha^{\prime }$ martensite in this study—at intersections of ϵ martensite, on ϵ martensite, and directly within austenite grains—are partially consistent with previous findings. For instance, Choi et al. and Xu et al. reported that martensite tends to nucleate at grain boundaries, shear bands, and twin interfaces in uniaxially loaded 304 and duplex steels [29,30]. However, the present study reveals that, under cyclic loading, the ϵ → $\alpha^{\prime }$ pathway plays a more dominant role than previously reported, especially at higher cycle numbers. Additionally, the identification of <101> orientation as the most transformation-prone under cyclic conditions adds new insights, which were not emphasized in prior work focused primarily on monotonic deformation. These differences highlight the importance of cyclic loading in modifying transformation pathways and orientation dependence.

## 4. Conclusions

In this study, the martensitic transformation behavior and its microscopic mechanism of Mn–N alloyed TRIP duplex stainless steel under cyclic loading were comprehensively investigated through in situ measurement, transformation kinetics modeling, and multiscale microstructural analysis. By employing a combination of experimental platforms and advanced characterization techniques, this work provides a systematic understanding of the strain-induced phase transformation under cyclic deformation, which is essential for the design of fatigue-resistant structural materials.

(1) Under cyclic loading, the metastable austenite does not completely transform into martensite at fatigue, and most metastable austenite is retained. The martensitic transformation increases with the increase in strain amplitude at the same cycle. The larger the strain amplitude is, the earlier the martensitic transformation begins. Under the cyclic loading, the martensitic transformation predominantly occurs in the initial 100 cycles, after which it gradually saturates.

(2) The transformation rate initially increases due to strain localization and active nucleation but decreases after approximately 15 cycles as transformable austenite is progressively consumed. Nevertheless, residual metastable austenite continues to transform gradually under ongoing cyclic strain, leading to a continued albeit slower increase in martensite content.

(3) A cyclic transformation kinetics model integrating the TI and Smaga models was proposed and calibrated based on experimental data. The model accurately predicts the evolution of martensite volume fraction under different strain amplitudes, with over 90% correlation with experimental results.

(4) The microscopic observations reveal three distinct nucleation mechanisms of $\alpha^{\prime }$ martensite: nucleation at the intersection of ϵ martensite, on ϵ martensite, and directly within the austenite matrix. This confirms the coexistence of both ϵ →$\alpha^{\prime }$ and γ→$\alpha^{\prime }$ transformation pathways under cyclic loading.

(5) Grain orientation significantly influences the transformation behavior. The austenite grain with <101> orientation is most susceptible to transformation, followed by <001> and <111>. The orientation evolution correlates well with transformation kinetics and explains the progressive transformation front during cycling.

Overall, this work not only clarifies the complex phase transformation behavior of Mn–N alloyed TRIP duplex stainless steel under cyclic loading, but also contributes a validated kinetics model and mechanistic understanding at both meso- and micro-scales. The findings offer theoretical guidance for material optimization in engineering applications such as automotive chassis and structural components, where strength, ductility, and cyclic stability are critical. Future work will focus on linking transformation behavior with fatigue damage accumulation and mechanical degradation under varying service conditions.

## Figures and Tables

**Table 1:** Chemical composition of the experimental steel (wt%).

C	N	Cr	Mn	Ni	Si	Mo	W	Fe
0.045	0.191	19.33	2.7	1.92	1.8	0.52	0.44	Bal.

**Table 2:** Fitting values of material constants.

Material Constants	k	Φ	m
Fitting value	−4	0.502	1.2

## Data Availability

The original contributions presented in this study are included in the article. Further inquiries can be directed to the corresponding authors.
